# The correlation between OSA-related quality of life and two mental statuses in adolescent Chinese patients with cleft palate: A comprehensive study

**DOI:** 10.3389/fped.2022.985375

**Published:** 2022-10-21

**Authors:** Yuan Zong, Xu Cheng, Weiyao Xia, Zhuojun Xie, Yichun Yang, Bing Shi, Caixia Gong, Hanyao Huang

**Affiliations:** State Key Laboratory of Oral Diseases and National Clinical Research Center for Oral Diseases and Department of Oral Maxillofacial Surgery, West China Hospital of Stomatology, Sichuan University, Chengdu, China

**Keywords:** cleft palate, OSA-related QoL, sleep-disordered breathing, anxiety, depression, OSA-18, GAD-7, PHQ-9

## Abstract

**Objectives:**

To analyze obstructive sleep apnea (OSA)-related quality of life (QoL), the statuses of depression and anxiety, and to reveal the correlation between OSA-related QoL and two mental statuses in Chinese adolescent patients with cleft palate (CP).

**Methods:**

The Obstructive Sleep Apnea Questionaire-18 (OSA-18), the Generalized Anxiety Disorder Scale (GAD-7) and the Patient Health Questionnaire-9 (PHQ-9) were applied to assess OSA-related QoL and the statuses of anxiety and depression in Chinese adolescent patients with CP, respectively. Non-CP adolescents were also included in the control group. OSA-related QoL and the two mental statuses were compared between the study and control groups. The correlation between the OSA-related QoL and two mental statuses was estimated in Chinese adolescent patients with CP.

**Results:**

A total of 8.7% patients showed a moderate or high impact of OSA on QoL, while all the adolescents from the control group showed little impact. The mean total OSA-18 score of the study group (36.261 ± 13.500) was significantly higher than the control (28.435 ± 8.934). The mean PHQ-9 scores of the study group and the control group were statistically different (3.957 vs. 2.113). The GAD-7 score in the study group was slightly higher than the control group (3.043 vs. 2.194), while the proportion of moderate-severe anxiety in the study group was relatively larger than that in the control group (6.5% vs. 1.6%). Moreover, there was a positive correlation between the OSA-related QoL and the statuses of anxiety and depression respectively, and the differences in GAD-7 and PHQ-9 scores between the moderate or high impact group and the little impact group were statistically significant.

**Conclusion:**

Chinese adolescents with CP reported a rate of moderate or high impact of OSA on QoL of 8.7%, which was significantly higher than adolescents without CP. The OSA-related QoL was worse and depression was severer in Chinese CP adolescents than in the control, while anxiety and depression in Chinese CP adolescents were associated with OSA-related QoL.

## Introduction

Cleft palate (CP) is one of the most common congenital abnormalities, which can lead to dysfunction of speech, breathing, and swallowing of the patients due to the abnormal anatomical structure of the palate and pharynx (1). CP might also bring nasal deformities, retrusive midface or small pharyngeal airway as the patients growing up, and herein exposes them to a high risk of obstructive sleep apnea (OSA) (2). It was found that the posterior airway space in adolescent patients after palatoplasty showed certain characteristics of OSA (3), which could affect the sleep-related breathing and related quality of life (QoL).

Sleep-disordered breathing (SDB), like OSA, could bring neuropsychiatric effects and adverse health consequences (4, 5), which could greatly affect the QoL of patients. It was reported that OSA had a correlation with mental disorders like anxiety and depression (6). Besides, it was also shown that anxiety and depression could affect sleep (7). Although sleep-related breathing problems had been found in patients with CP (8), their OSA-related QoL had not received due attention, and there was a lack of relevant study in Chinese patients with CP.

In this study, we sought to compare the OSA-related QoL, and the statuses of depression and anxiety, between patients and control adolescents, and ulteriorly demonstrate the correlation between the OSA-related QoL and these two mental statuses, anxiety and depression. The Obstructive Sleep Apnea Questionaire-18 (OSA-18) has been widely used to assess QoL in children with SDB in previous studies, in children with macroglossia and adenotonsillar hypertrophy for example (9, 10), and therefore was considered as a valid measure of health-related QoL (11). In our study, OSA-18 was applied to assess the QoL in children with CP, while the generalized anxiety disorder 7-item (GAD-7) scale and the Patient Health Questionnaire-9 (PHQ-9) were applied to assess the statuses of anxiety and depression. Based on this study, we tried to provide a theoretical basis for cleft palate care and possible psychological intervention.

## Materials and methods

### Subjects

Chinese Patients with a history of cleft palate hospitalized in West China Hospital of Stomatology, Sichuan University, between July 2019 and January 2021 were incorporated in the study group.

Inclusion criteria of the study group were as follows:

(1) Patients with cleft palate;
(2) Patients who had no other syndromic diseases;
(3) Patients who underwent palatoplasty at the age of 6 months to 2 years;
(4) Patients without secondary surgery of cleft palate or other types of speech surgery like posterior pharyngeal flap or orthognathic surgery at the time of data collecting;
(5) Patients aged above 10 (12) and under 17 (13).

Inclusion criteria of the control group were as follows:

(1) Individuals who had cone-beam computed tomography scan for other dental treatments;
(2) Individuals with normal speech and without significant nasal septal deviation or micromandible or other significant airway diseases;
(3) Individuals aged above 10 and under 17.

A total of 46 patients were enrolled (Mean age: 13.41 ± 2.14 years), while a total of 62 normal adolescents were enrolled (Mean age: 13.84 ± 0.91 years). Among the study group, 63.0% were males; 87.0% had cleft lip and palate, and 13.0% had cleft palate only; 26.1% were diagnosed with velopharyngeal insufficiency. In the control group, 54.8% were males.

The study was approved by the Ethics Committee of West China Hospital of Stomatology, Sichuan University (No. WCHSIRB-D-2016-084R1). All the parents of the patients enrolled in the study provided written informed consents.

### Assessment of OSA-related QoL

The Obstructive Sleep Apnea Questionaire-18 (OSA-18) was used to assess the OSA-related QoL, which contained 18 items rated from 1 to 7 with a Likert scale. The 18 items were grouped into 5 domains: sleep disturbance, physical suffering, emotional distress, daytime problems, and caregiver concerns. A total score above 60 suggested that the sleep disorder had a moderate or high impact on QoL (14). Good reliability and validity of Chinese-version OSA-18 have been validated in patients aged 2–18 years old (15), and OSA-18 has been used in adolescent patients with cleft palate (2). The questionnaire was filled in by the patients' parents or legal guardians independently based on the situation of their children in the past 4 weeks (16).

### Assessment of anxiety

The Generalized Anxiety Disorder 7-item (GAD-7) scale was given to adolescents themselves to assess their anxiety status over the last 2 weeks with 7 items graded on a 4-point Likert scale as 0 (not at all), 1 (several days), 2 (more than half the days), and 3 (nearly every day). The subjects were divided into normal (0–4), mild (5–9), moderate (10–14) and severe (15–21) according to the total score. Chinese-version GAD-7 has been widely used in Chinese population, notably in the cleft field (17), and has shown good reliability and validity among Chinese adolescents aged 10–17 years old (12).

### Assessment of depression

The Patient Health Questionnaire-9 (PHQ-9) was applied to assess depression status in the past 2 weeks, which consisted of 9 items on a 4-point scale ranging from 0 (not at all) to 3 (nearly every day). A total score of 10 points was considered to be the dividing line between depression and non-depression. PHQ-9 has been widely used to assess depressive symptoms in patients with specific diseases (18–20), and the reliability and effectiveness of PHQ-9 have been validated in Chinese adolescents aged 11–17 years old (21), and has been applied to autistic patients aged 10–18 in Singapore (22).

In this study, all subjects received questionnaires of the Chinese version and to ensure that each item was fully understood, questionnaires were completed under the guidance of trained volunteers.

### Statistical analysis

Statistical analysis was performed using SPSS 26.0 (IBM Corp., Armonk, NY, USA), and count data were expressed as the mean ± standard deviation (SD). Mann–Whitney *U* test was used to compare the two independent variables between groups, and categorical variables (impact on QoL) were compared between the groups using Fisher exact test, while Spearman's rank correlation was used to estimate the correlation among the OSA-related QoL, anxiety status and depression status. Shapiro-Wilk demonstrated non-normal distribution of those statistics. ***P***-values <0.05 were statistically significant.

## Results

### OSA-related QoL in the study and control groups

The mean total OSA-18 scores of the study and control groups were 36.261 and 28.435 points respectively, and differences in total OSA-18 score, domains including emotional distress, daytime problems and caregiver concern and several items of OSA-18 (OSA-18-8/10/12/15/16/17/18) were statistically significant, among which the mean score of the study group was higher than that of the control group (Table 1). 8.7% of the total OSA-18 scores were above 60 in the study group, showing moderate or high impact on QoL, while all the adolescents from the control group showed little impact, which was statistically significant (Table 2).

**Table 1:** The OSA-18 scores of the study group and control group.

Variable	Mean ± SD		*P*-value
	The study group	The control group	(Study vs. Control)
OSA-18 components			
Total OSA-18 score	36.261 ± 13.500	28.435 ± 8.934	0.001**
Sleep disturbance	5.674 ± 2.271	5.161 ± 1.857	0.168
OSA-18-1: Loud snoring	1.565 ± 0.958	1.339 ± 0.767	0.143
OSA-18-2: Breath holding/pauses	1.152 ± 0.666	1.145 ± 0.507	0.591
OSA-18-3: Choking or gasping	1.217 ± 0.593	1.226 ± 0.638	0.728
OSA-18-4: Fragmented sleep	1.739 ± 1.084	1.452 ± 0.783	0.176
Physical suffering	6.848 ± 3.326	6.081 ± 2.706	0.227
OSA-18-5: Mouth breathing	1.717 ± 1.109	1.887 ± 1.415	0.766
OSA-18-6: Frequent colds or URIs	1.783 ± 1.246	1.597 ± 0.999	0.500
OSA-18-7: Rhinorrhea	2.000 ± 1.333	1.581 ± 1.001	0.075
OSA-18-8: Dysphagia	1.348 ± 0.737	1.016 ± 0.127	0.001***
Emotional distress	5.587 ± 2.325	4.790 ± 2.362	0.022*
OSA-18-9: Mood swings or tantrums	2.130 ± 1.240	1.952 ± 1.324	0.23
OSA-18-10: Aggression/hyperactivity	1.870 ± 1.185	1.452 ± 1.003	0.009**
OSA-18-11: Discipline problems	1.587 ± 1.002	1.387 ± 0.610	0.508
Daytime problems	7.196 ± 3.976	5.677 ± 2.597	0.048*
OSA-18-12: Daytime drowsiness	1.913 ± 1.411	1.355 ± 0.630	0.013*
OSA-18-13: Poor attention span	2.674 ± 1.752	2.194 ± 1.502	0.136
OSA-18-14: Difficulty awakening	2.609 ± 1.807	2.129 ± 1.152	0.326
Caregiver concern	10.957 ± 5.617	6.726 ± 3.825	0.000***
OSA-18-15: Caregiver worried about child's health	4.239 ± 2.110	2.371 ± 1.776	0.000***
OSA-18-16: Caregiver concerned child does not have enough air	2.783 ± 2.250	1.645 ± 1.427	0.005**
OSA-18-17: Caregiver missed activities	1.935 ± 1.652	1.306 ± 0.781	0.028*
OSA-18-18: Caregiver frustration	2.000 ± 1.700	1.403 ± 0.983	0.027*

* *P* < 0.05, **P < 0.01, ***P < 0.001 by Mann–Whitney *U* test.

**Table 2:** Comparison of the impact of OSA on QoL between the study group and control group.

	The study group	The control group	*P*-value
Moderate or high impact	4 (8.7%)	0 (0%)	0.030*
Little impact	42 (91.3%)	62 (100%)	
Total	46	62	

*P < 0.05 by Fisher's Exact Test.

### Anxiety status in the study and control groups

There was no statistically significant difference in GAD-7 total score and scores of each item between the study group and the control group. However, the mean GAD-7 total score in the study group was slightly higher than the control group (3.043 vs. 2.194) (Table 3). Besides, the proportion of moderate-severe anxiety in the study group was larger than in the control group (6.5% vs. 1.6%) (Table 4).

**Table 3:** The GAD-7 scores of the study group and control group.

Variable	Mean ± SD		*P*-value
	The study group	The control group	(Study vs. Control)
Total GAD-7 score	3.043 ± 3.608	2.194 ± 2.851	0.080
GAD-7-1: Feeling nervous, anxious or on edge	0.565 ± 0.620	0.387 ± 0.554	0.104
GAD-7-2: Not being able to stop or control worrying	0.304 ± 0.628	0.306 ± 0.561	0.751
GAD-7-3: Worrying too much about different things	0.391 ± 0.774	0.387 ± 0.554	0.445
GAD-7-4: Trouble relaxing	0.370 ± 0.572	0.306 ± 0.531	0.553
GAD-7-5: Being so restless that it is hard to sit still	0.348 ± 0.737	0.210 ± 0.410	0.597
GAD-7-6: Becoming easily annoyed or irritable	0.696 ± 0.840	0.419 ± 0.588	0.103
GAD-7-7: Feeling afraid as if something awful might happen	0.370 ± 0.679	0.177 ± 0.385	0.152

**Table 4:** Severity of anxiety in the study and control groups.

	Total	Normal		Mild anxiety		Moderate and severe anxiety		
		Number	Percentage	Number	Percentage	Number	Percentage
The study group	46	38	82.60%	5	10.90%	3	6.50%
The control group	62	49	79.00%	12	19.40%	1	1.60%

### Depression status in the study and control groups

The mean total PHQ-9 scores of the study group and the control group were statistically different (3.957 vs. 2.113), and the mean scores of items “Poor appetite or overeating” and “Feeling bad about yourself or that you are a failure or have let yourself or your family down” showed statistical difference (Table 5).

**Table 5:** The PHQ-9 scores of the study group and control group.

Variable	Mean ± SD		*P*-value
	The study group	The control group	(Study vs. Control)
Total PHQ-9 score	3.957 ± 4.685	2.113 ± 2.931	0.018*
PHQ-9-1: Little interest or pleasure in doing things	0.435 ± 0.720	0.306 ± 0.465	0.552
PHQ-9-2: Feeling down, depressed, or hopeless	0.391 ± 0.649	0.306 ± 0.499	0.703
PHQ-9-3: Trouble falling or staying asleep, or sleeping too much	0.652 ± 0.948	0.274 ± 0.485	0.053
PHQ-9-4: Feeling tired or having little energy	0.478 ± 0.752	0.258 ± 0.441	0.191
PHQ-9-5: Poor appetite or overeating	0.630 ± 0.799	0.290 ± 0.524	0.018*
PHQ-9-6: Feeling bad about yourself or that you are a failure or have let yourself or your family down	0.609 ± 1.000	0.226 ± 0.525	0.040*
PHQ-9-7: Trouble concentrating on things, such as reading the newspaper or watching television	0.283 ± 0.688	0.145 ± 0.355	0.562
PHQ-9-8: Moving or speaking so slowly that other people could have noticed? Or the opposite – being so fidgety or restless that you have been moving around a lot more than usual	0.326 ± 0.732	0.177 ± 0.497	0.399
PHQ-9-9: Thoughts that you would be better off dead or of hurting yourself in some way	0.152 ± 0.420	0.129 ± 0.338	0.949

* *P* < 0.05 by Mann–Whitney *U* test.

### Correlation analysis between the OSA-related QoL and the statuses of anxiety and depression

There was a positive correlation between the OSA-related QoL and the statuses of anxiety and depression, respectively. The total OSA-18 score and domains including sleep disturbance, emotional distress and caregiver concern were all positively correlated with GAD-7 scores, while the total OSA-18 score and domains including sleep disturbance, emotional distress, daytime problems and caregiver concern were positively correlated with PHQ-9 scores (Table 6).

**Table 6:** Correlation analysis among the OSA-18, GAD-7 and PHQ-9 in the study group.

Variable	GAD-7		PHQ-9	
	*ρ*	*P*-value	*Ρ*	*P*-value
OSA-18 components				
Total OSA-18 score	0.339	0.021*	0.461	0.001**
Sleep disturbance	0.317	0.032*	0.503	0.000***
Physical suffering	0.138	0.359	0.289	0.051
Emotional distress	0.307	0.038*	0.394	0.007**
Daytime problems	0.250	0.094	0.457	0.001**
Caregiver concern	0.333	0.024*	0.389	0.008**

* *P* < 0.05, ** *P* < 0.01, ***P < 0.001 by Spearman's rank correlation.

### The analysis of the impact situations of OSA-related QoL on mental status

The differences of GAD-7 and PHQ-9 scores between the moderate or high impact group and the little impact group were statistically significant, which demonstrated the influence of OSA-related QoL on these two mental statuses (Table 7).

**Table 7:** The GAD-7 and PHQ-9 scores of different impact situations of OSA-related QoL in the study group.

Variable	Mean ± SD		*P*-value
	Moderate or high impact	Little impact	
GAD-7	7.000 ± 4.690	2.667 ± 3.318	0.028*
PHQ-9	10.250 ± 4.992	3.357 ± 4.247	0.014*

* *P *< 0.05 by Mann–Whitney *U* test.

## Discussion

Cleft palate may lead to nasal deformities, retrusive midface or small pharyngeal airway, which bring a high risk of OSA (2). OSA-related symptoms, such as snoring and noisy breathing during sleep were often reported in children with CP (3). Even after palatoplasty, upper airway dimensions were still reduced in patients with CP (23). OSA had a significant impact on quality of life, which could lead to anxiety and depression (24, 25) and result in adverse societal and individual correlates (26). In this study, we aimed to analyze OSA-related QoL and the statuses of depression and anxiety, and to further reveal the correlation between OSA-related QoL and two mental statuses in Chinese adolescent patients with CP.

According to OSA-18 results, we found that the average OSA-18 score of Chinese adolescent patients with CP was higher than the controls, which might reveal that adolescents with CP were at higher risk of OSA, consistent with the results evaluated by the Pediatric Sleep Questionnaire (PSQ) and polysomnography (PSG) in the previous studies (27, 28). The rate of moderate or high impact of OSA on QoL in children with CP (8.7%), which was approximately consistent with previous studies (9.5%) (2), was significantly higher than that of the control group (0%). As for the statistically significant domains of emotional distress, daytime problems and caregiver concern in the control group, we found that the influence of OSA on QoL in patients with CP was more manifested in emotion, behavior and the influence on caregivers than the physical symptoms, which coincided with previous studies in children with CP (29–31). Compared with non-cleft, the clinical symptoms of OSA in children with CP was similar (29). However, aggressive behavior and defiant temper were confirmed in children with CP under the influence of sleep-disordered breathing (30), and daytime functional problems such as daytime sleepiness and inattention were reported in children with CP in previous studies (31).

Our research further confirmed these conditions not only in children but also in adolescents with CP. As for the caregiver concern dimension, the sleep-disordered breathing problems of children with CP were often reported by their parents (3), and under greater pressure, the quality of life and psychology of parents of children with CP were affected simultaneously (32).

Our results showed that 6.5% of adolescent patients with CP were in moderate or severe anxiety, compared with 1.6% in the control group. However, no significant difference in total GAD-7 scores and scores of each item between the study group and the control group was found, which was consistent with the results of previous studies that there was no significant difference in life satisfaction and anxiety between patients with CP and the non-CP control group (17, 33), but the proportion of patients with CP reporting anxiety problems was higher than that of the control group (34).

In contrast to anxiety status, there were significant differences in total PHQ-9 scores screening depression between the study group and the control group. As some studies have shown, compared with healthy children, depression in children with CP was more common (35). Items that demonstrated statistically significant difference were “Poor appetite or overeating” and “Feeling bad about yourself or that you are a failure or have let yourself or your family down”. Patients with CP had difficulty chewing, especially biting hard food (36), and showed a more negative self-awareness (37), which might explain why the study group showed higher scores than the control in those items.

It used to be thought that the anxiety and depression of patients with CP mainly came from their abnormal appearance and pronunciation (38). However, our study reported that OSA-related QoL were also associated with anxiety and depression in Chinese adolescent patients with CP. Beyond this, the statuses of anxiety and depression between the moderate or high impact group and the little impact group of OSA-related QoL showed statistical significance. Previous studies have confirmed the relationship between OSA and the statuses of anxiety and depression (6, 39), and the same conclusion was drawn in Chinese adolescent patients with CP in our study.

The results showed that anxiety and depression were correlated with a number of domains of OSA-related QoL. However, there was no significant correlation between the physical suffering dimension and the statuses of anxiety and depression, which might be interpreted as, compared to the discomfort caused by sleep breathing symptoms, the psychological impact of CP was more from the mental and behavioral problems caused by it. The emotional distress dimension of OSA-18 scale was significantly correlated with GAD-7 and PHQ-9 scales, which reflected the homogeneity of the three scales in evaluating psychological status. It was also found that the caregiver concern dimension had significant correlation with GAD-7 and PHQ-9 scales. Other studies have shown that the life quality of parents of children with CP were relatively poor (32), while this study showed that this influence may be mutual. The stress and worries from parents were also related to the psychological status of children, social support and correct coping strategies being beneficial to alleviate this influence (40). Interestingly, depression symptoms were uniquely associated with daytime problems domain, which meant that children's OSA-related negative behavior during the day led to depression rather than anxiety. It might be explained that sleep may have etiologically distinct and directional associations with anxiety and depression (41).

There were some limitations in our study. The sample size should be enhanced in the future. This study was conducted only in a stomatological hospital in western China. Also, no conclusion could be drawn on the direction of causality because of the study type of cross-sectional study, and there was only scale measurement focusing on QoL, lacking objective sleep measurement. Another limitation that should be mentioned is that OSA-18 is a caregiver-administered tool based on targeted discussions with caregivers of children with OSA (16), but studies have shown discrepancies in patients vs. parents' perceptions of their conditions (42–44). Thus, further study is needed with a suitable patient-administered tool for screening OSA.

## Conclusion

Chinese adolescents with CP reported a prevalence of rate of moderate or high impact of OSA on QoL of 8.7%, which was significantly higher than adolescents without CP. The OSA-related QoL was worse and depression was severer in Chinese CP adolescents than the control. Anxiety and depression in Chinese CP adolescents were associated with OSA-related QoL.

## Data Availability

The original contributions presented in the study are included in the article/Supplementary Material, further inquiries can be directed to the corresponding authors.
